# Supplementary material for: Delivering equity in low-carbon multisector infrastructure planning
Source: Nat Commun. 2025 Jun 19;16:5320. doi: 10.1038/s41467-025-59738-7 (PMC12179266; doi:10.1038/s41467-025-59738-7)
Supplement: Supplementary file 1 — Supplementary Information [file 41467_2025_59738_MOESM1_ESM.pdf]

Supplemental Information

Delivering equity in low-carbon multisector infrastructure planning

Adil Ashraf [1], Mohammed Basheer [1, 2, 3], Jose M. Gonzalez [1], Eduardo A. Martínez Ceseña [4, 5], Mikiyas Etichia [1], Emmanuel Obuobie [6], Andrea Bottacin-Busolin [7, 1], Jan Adamowski [8], Mathaios Panteli [4, 9] & Julien J. Harou [1, 10, *]

[1] Department of Civil Engineering and Management, The University of Manchester, Manchester, UK
[2] Department of Civil and Mineral Engineering, University of Toronto, Toronto, Canada
[3] Thaer-Institute of Agricultural and Horticultural Sciences, Humboldt University of Berlin, Berlin, Germany
[4] Department of Electrical and Electronic Engineering, The University of Manchester, Manchester, UK
[5] Tyndall Centre for Climate Change Research, The University of Manchester, Manchester, UK
[6] Water Research Institute, Council for Scientific and Industrial Research, Accra, Ghana
[7] Department of Industrial Engineering, University of Padua, Padua, Italy
[8] Department of Bioresource Engineering, McGill University, Montreal, Canada
[9] Department of Electrical and Computer Engineering, University of Cyprus, Nicosia, Cyprus
[10] Department of Civil, Environmental and Geomatic Engineering, University College London, London, UK

* Correspondence to: julien.harou@manchester.ac.uk

This document includes:

- Supplementary Figure 1 to Figure 5
- Supplementary Table 1
- Supplementary References

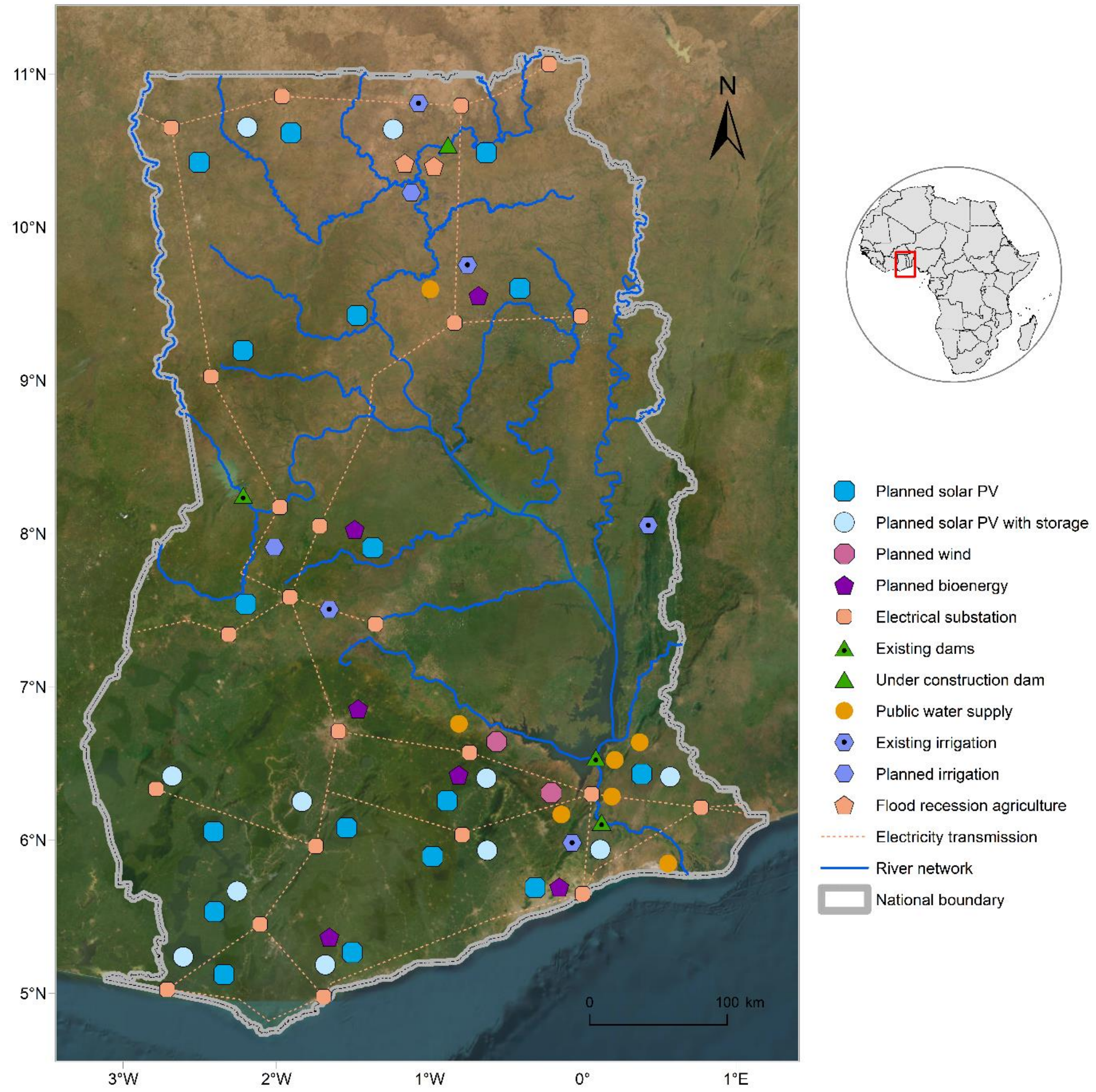

Supplementary Figure 1: Ghana's river network and power system infrastructure. This map shows the electrical transmission network; planned solar photovoltaic (PV), combined solar PV with storage, wind power, and bioenergy generation sites; existing and under construction storage dams; municipal water supply diversions; existing and planned irrigation schemes; and flood recession agriculture activities in Ghana. The river and power system infrastructure data are from the Ghana Council for Scientific and Industrial Research - Water Research Institute, the Ghana Grid Company, and previous publications on the Ghana simulation model[1,2]. The national boundary layer is based on data from Ghana Statistical Services (GSS). The background satellite image is based on the imagery of the world managed by the Environmental Systems Research Institute (ESRI).

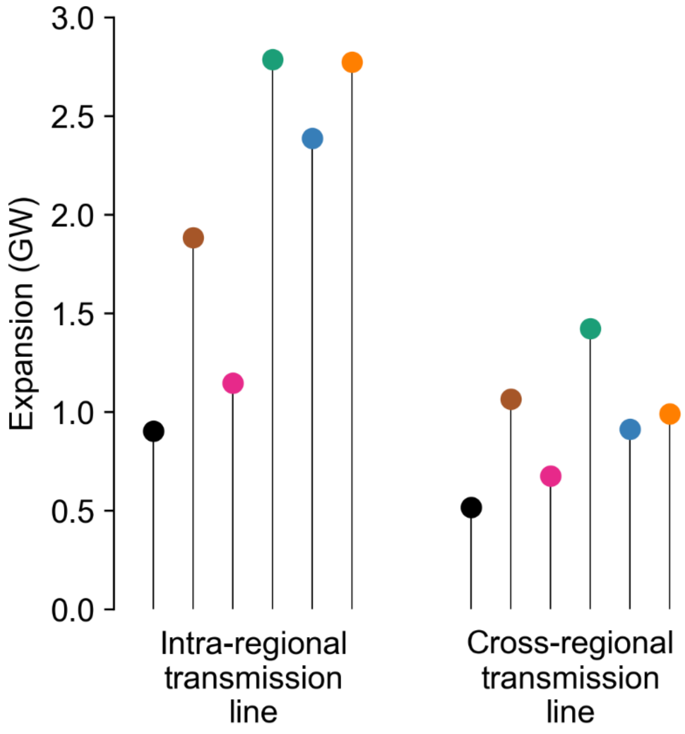

Supplementary Figure 2: Intra- and cross-regional electrical transmission line expansion for Ghana of the selected efficient portfolios of Fig. 3a. This figure shows that the intra-regional transmission line expansion is higher than cross-regional transmission line expansion for the baseline and selected Pareto-efficient portfolios. The circle colours correspond to the lines with the same colours in Fig. 3a. GW stands for gigawatts. Source data are provided as a Source Data file.

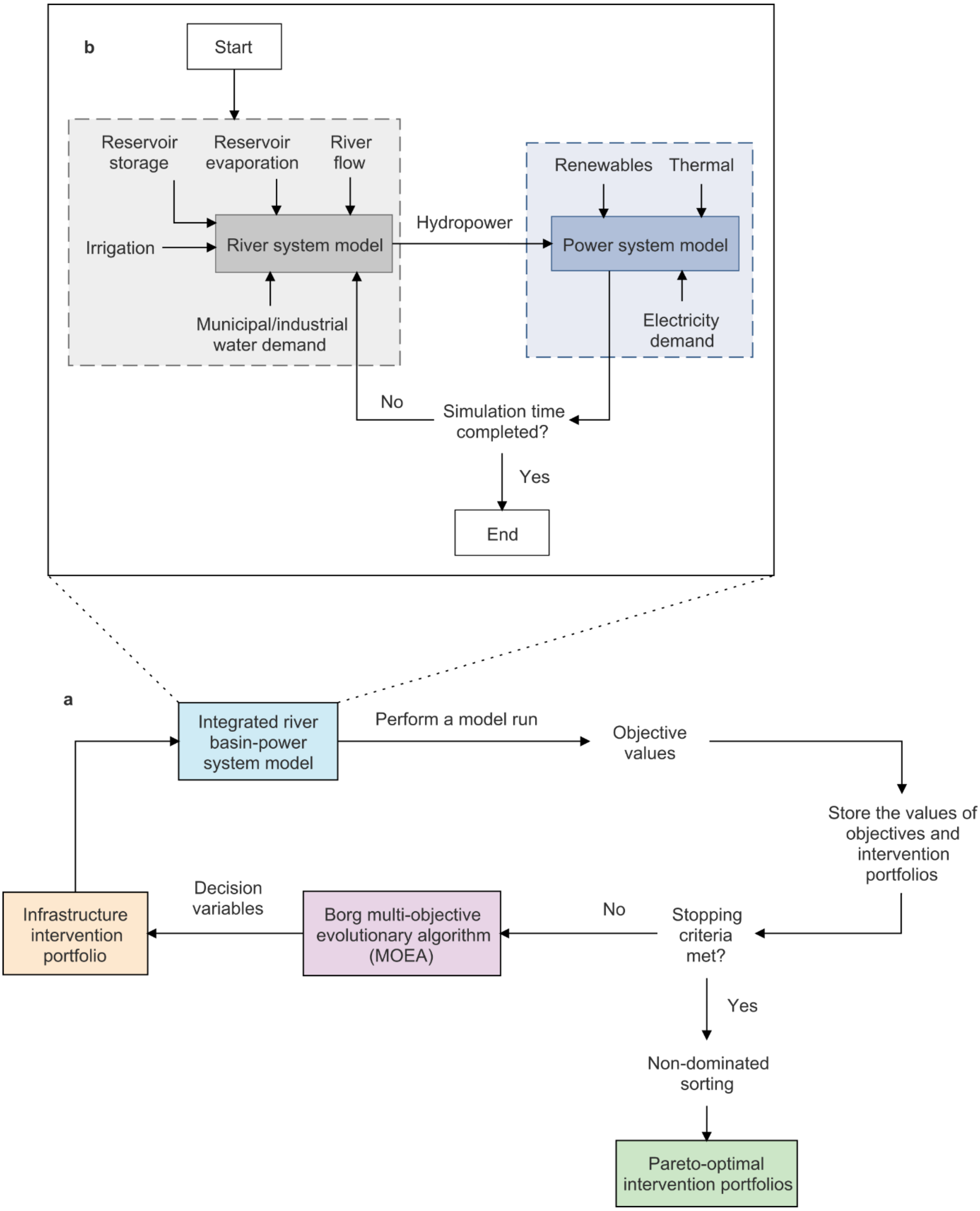

Supplementary Figure 3: Flowchart on the iteration between the integrated river basin-power system model and the multi-objective evolutionary algorithm (MOEA) for identifying non-dominated, approximately Pareto-optimal infrastructure intervention portfolios. a) Simulation-based artificial intelligence-assisted multi-objective design process, b) Integrated river basin-power system simulation model.

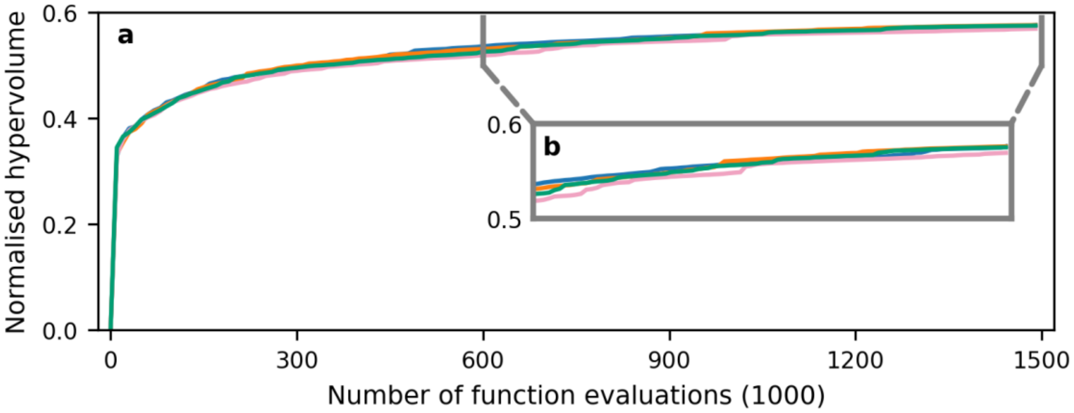

Supplementary Figure 4: Evolution of the hypervolume for four random seeds. a) Hypervolume over all function evaluations, b) Hypervolume between 600 and 1500 function evaluations. Each random seed represents a unique starting point for the search algorithm and is shown by a different coloured line in this figure. Source data are provided as a Source Data file.

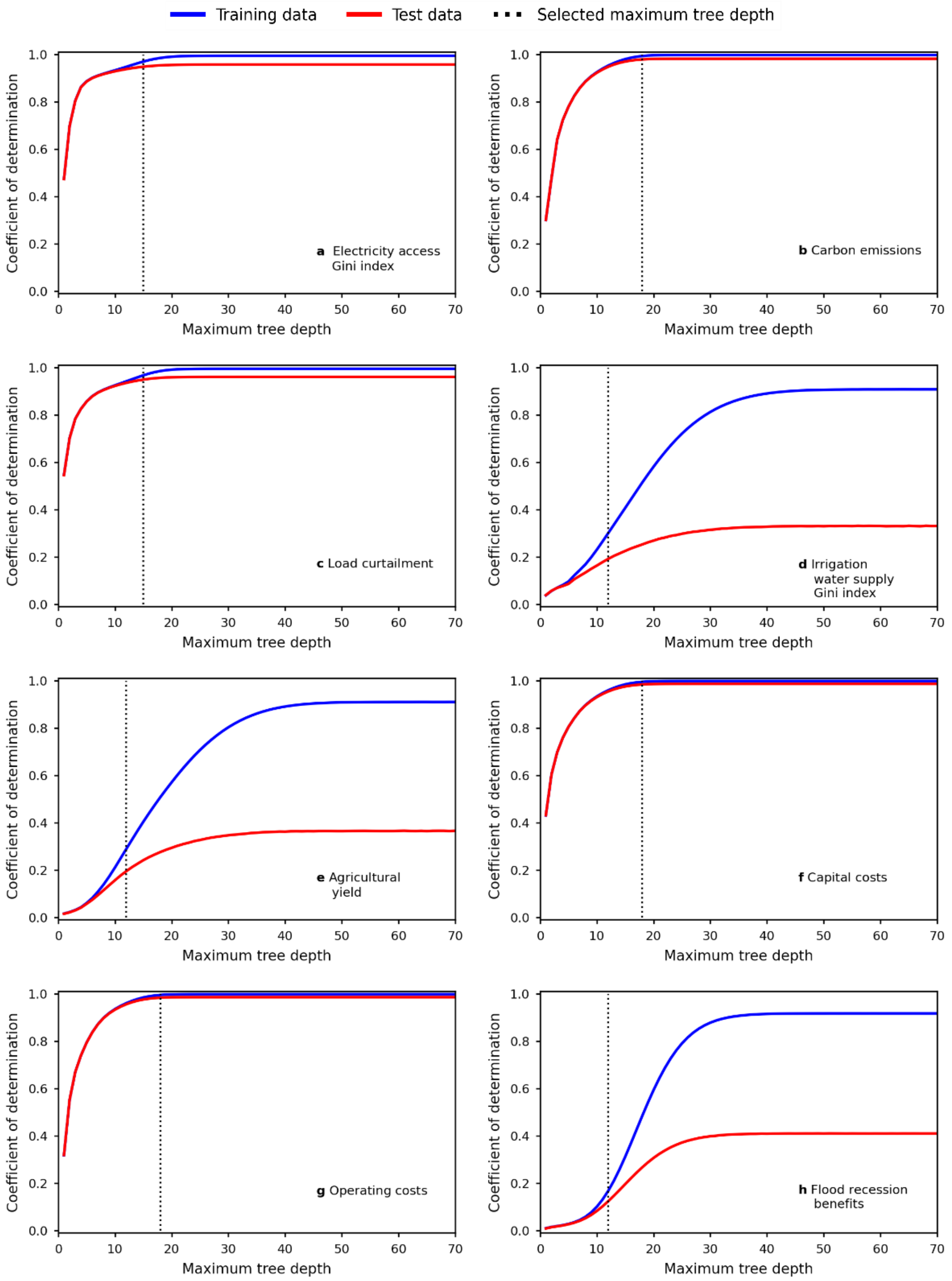

Supplementary Figure 5: Performance of the Random Forest Regression machine learning models for the training and testing datasets with maximum tree depths ranging from 1 to 70. This figure shows the performance of the machine learning models in predicting the integrated water-energy system performance indicators for different maximum tree depths for the training and testing datasets. 80% of the data was used for training and 20% of the data was used for testing. A best-fit tree depth was selected for each model to prevent overfitting or underfitting the data. Source data are provided as a Source Data file.

Supplementary Table 1: Type and number of nodes in the Ghana multisector infrastructure system simulation model.

Model	Node type	Number of nodes
River system	Catchment inflow	9
	Reservoir	9
	Hydropower*	6
	Municipal water supply	5
	Irrigation	10
	Flood recession farming	1
	Flood recession pond fishing	1
	Environmental flow	1
Power system	Solar	19
	Combined solar and storage	11
	Wind power	2
	Bioenergy	7
	Hydropower	4
	Thermal generators	24
	Substations	23
	Transmission lines	33
	Electricity demand	22
*These are hydropower nodes within the Volta River basin, including those located in Ghana.		

Supplementary References

1. Gonzalez, J. M. *et al.* Quantifying cooperation benefits for new dams in transboundary water systems without formal operating rules. *Front. Environ. Sci.* **9**, 596612 (2021).
2. Gonzalez, J. M. *et al.* Designing diversified renewable energy systems to balance multisector performance. *Nat. Sustain.* **6**, 415–427 (2023).
